# Efficacy of Desvenlafaxine in Reducing Migraine Frequency and Severity: A Retrospective Study

**DOI:** 10.3390/jcm13175156

**Published:** 2024-08-30

**Authors:** Marina Stoupa Hadidi, Murad Rasheed, Yanal M. Bisharat, Heba H. Al Helou, Hussam A. El Aina, Hala M. Batayneh, Alaa A. A. Aljabali, Omar Gammoh

**Affiliations:** 1 Jordan Hospital, Adib Wehbeh St., Amman 11152, Jordan; marinahadidi@gmail.com; 2 The Specialty Hospital, Hunayn Bin Ishak St, Amman 11193, Jordan; murad_rasheed@yahoo.com; 3 Medical Affairs Department, MS Pharma Regional Office, Zahran Plaza Bldg., 7th Circle Amman, Amman 11844, Jordan; yanal.bisharat@mspharma.com (Y.M.B.); heba.alhelou@mspharma.com (H.H.A.H.); 4 Marketing Department, MS Pharma Regional Office, Zahran Plaza Bldg., 7th Circle Amman, Amman 11844, Jordan; hussam.alaina@mspharma.com (H.A.E.A.); hala.batayneh@mspharma.com (H.M.B.); 5 Department of Pharmaceutics and Pharmaceutical Technology, Yarmouk University, Irbid 21163, Jordan; alaaj@yu.edu.jo; 6 Department of Clinical Pharmacy and Pharmacy Practice, Faculty of Pharmacy, Yarmouk University, Irbid 21163, Jordan

**Keywords:** desvenlafaxine, migraine, prophylaxis, pain, women, episodes, headache, frequency, severity, disability

## Abstract

**Background:** Migraine is characterized by sudden acute episodes of pain, with a global prevalence of 18% among all age groups. It is the second leading cause of years lived with disability worldwide. Prophylactic treatment is important in managing migraine; however, its efficacy and safety are debated. This study aimed to evaluate the efficacy of desvenlafaxine in female patients with migraine. **Methods:** We conducted a retrospective observational case study involving 10 women diagnosed with migraine who were treated with desvenlafaxine. We measured the number of migraine days per month, average headache duration in minutes, headache severity using a visual analog scale, use of acute medications, and frequency of acute medication use per week. **Results:** Desvenlafaxine significantly reduced the number of migraine days from 14.70 ± 3.68 at baseline to 2.50 ± 2.50 at follow-up (*p* < 0.05). The average headache duration dropped from 131.25 ± 32.81 min to 52.50 ± 44.64 min. Headache severity scores improved from 6.80 ± 1.49 at baseline to 0.80 ± 0.92 at follow up, the frequency of acute medication use per week reduced from 3.30 ± 1.49 at baseline to 0.80 ± 0.92, and the frequency of acute medication use decreased from 3.30 ± 1.49 times per week to 0.80 ± 0.92. **Conclusions:** Desvenlafaxine shows potential as an effective prophylactic therapy for migraine. Larger-scale studies are necessary to further explore its benefits.

## 1. Introduction

Migraine is a worldwide disabling neurological disorder featuring severe headaches that affects more than 10% of the general population [1]. Migraine presents in women more than in men; according to current estimations, 12% of women versus 6% of men are diagnosed with migraine [2], leading to professional and social disabilities [3]. Migraine attacks may last for hours or even days. They come with awful pain, nausea, and sensitivity to light and sound. This means losing workdays and time. Individuals suffering become less productive and experience impaired social and professional life.

The research on migraine in women is deeply rooted. Migraine was shown to almost always prevail in women at higher rates than men; for example, one study underscored that women were diagnosed with migraine twice as often as men [1,4]. Moreover, according to the estimations, migraine attacks were higher in females; several studies confirmed that women could suffer from more than one attack per month [1,5]. This fact also explains the higher number of physicians visit due to migraine; one study demonstrated that women account for almost 80% of migraine physicians visits [6].

According to the literature and clinical experience, people with migraine do not only suffer from the experience of migraines; the worst consequence is the continuous worry about the next migraine episode, including its intensity, timing, and duration. This presents these patients with a higher risk of psychological burden, which significantly affects their behavior and quality of life. For instance, some patients could have increased anxiety, social avoidance, and disruption of their social relationships and jobs [7,8]. According to the estimations of the Global Burden of Disease in 2016, migraine was labeled as the second leading cause of years lived with disability [9,10]. Therefore, the socioeconomic burden of migraine is an important nexus that warrants investigation. In Germany, for example, a recent analysis comprising >15 million migraine patients revealed that the annual socioeconomic losses for migraine are estimated to be EUR 100.4 billion [8]

According to the literature, migraine headaches and depression are highly associated [11,12]. This can be attributed to genetic factors, environmental factors, and stress, which activate catecholamines such as serotonin and norepinephrine. In general, the role of serotonin is evident in migraine headache pathophysiology; migraine is associated with low serotonin levels, especially between attacks [13].

Migraine pathophysiology represents a complex interplay between numerous neurotransmitters, proteins, enzymes, and genes. The role of serotonin (5-hydroxytryptamine (5-HT)) in migraine has been studied for decades. The serotonergic system projects nearly to all the different regions of the brain, including the sensory cortex, the thalamus, and the dorsal horns of the spinal cord, and is involved in many functions related to pain modulation, cortical sensory processing, and others based on its interaction with its different receptors [14,15,16,17,18]. Early evidence suggests the involvement of circulating serotonin in migraine. One very early investigation demonstrated that serotonin levels were significantly higher in people with migraine compared to their healthy peers during migraine attacks. These high levels, however, were normalized between attacks; this could be due to the intense enzymatic degradation by monoamine oxidases [19,20]. Even earlier evidence pointed towards an increased urinary excretion of a major metabolite of serotonin, 5-hydroxy indole acetic acid, during migraine attacks [13]. These clinical findings could establish a trend that highlights that people with migraine have low serotonin levels that spike during the attacks. In addition, in vivo studies have demonstrated a significant role of norepinephrine in the inhibition of neuropathic pain [21]. Central pain modulation is mediated by the midbrain periaqueductal grey matter. This pain modulation mechanism is stimulated by the enkephalin-releasing neurons that, in turn, activate the rostral ventromedial medulla, which in turn releases serotonin and norepinephrine, gamma-aminobutyric acid, and other mediators [22,23]. Mediations that can interfere with the release of these medications can have analgesic effects [22,24].

The endogenous descending pain inhibitory system, stemming from the rostral ventral medulla to the spinal cord, is mainly activated by norepinephrine [25,26]. Therefore, maintaining high post-synaptic levels of norepinephrine, and, to a lesser extent, serotonin, results in a sustained activation of the descending pain inhibitory pathway [27,28]. This can be achieved by using medications that inhibit the transporters of norepinephrine and serotonin such as tricyclic antidepressants and serotonin-norepinephrine reuptake inhibitors.

This supports the plethora of previous studies highlighting the roles of amitriptyline and venlafaxine in migraine prophylaxis, underscoring the implication of serotonin and norepinephrine in migraine [29,30]. Published studies indicate that dopamine and glutamate may contribute to migraine pathophysiology. This can also explain why people report very different symptoms. Very different triggers activate them. This is due to the complex interplay of these neurotransmitters [31,32].

The availability of a wide range of prophylactic therapy for patients with migraine is crucial because it improves the daily functioning and the quality of life of patients and reduces the consumption of acute analgesics and other related medications [33]. Despite the approval of several prophylactic therapies for migraine headaches, such as beta-blockers and antiepileptic medications, the evaluation of antidepressants such as serotonin-norepinephrine reuptake inhibitors (SNRIs) widens the therapeutic options for clinicians as SNRIs are preferred over TCAs due to their higher safety profile, mainly related to the cardiovascular and anticholinergic side effects [34]

The current prophylactic treatments for migraine include beta-blockers, antiepileptics, antidepressants, and biological treatments [35,36]. All these medications have their own efficacy profiles and side effects. These reported results show the need for diverse treatment options. Desvenlafaxine, a well-known potent and effective SNRI, is used in major depression disorder and demonstrated efficacy in improving neuropathic pain symptoms. Desvenlafaxine, the primary active metabolite of venlafaxine, is administered as desvenlafaxine succinate and is usually well-tolerated at a dose range of 50–100 mg/day [37]. Higher doses, reaching up to 400 mg/day, were still tolerated and effective in diabetic neuropathy [38,39]. According to the literature, no previous reports demonstrated the potential benefit of desvenlafaxine in improving migraine outcomes in female patients with migraine. One very recent study reported desvenlafaxine efficacy in migraine prophylaxis [40]. Therefore, the present case series aims to provide a preliminary evaluation of desvenlafaxine as a potential prophylactic therapy for females with migraine. Desvenlafaxine helps in treating depression and nerve pain. It has fewer side effects compared to tricyclic antidepressants. As such, it may be a good option for preventing migraines. This drug has two effects, acting on both the serotonin and norepinephrine systems. These imbalances mirror those seen in patients with migraine [41].

## 2. Materials and Methods

### 2.1. The Study Design and Settings

This is an observational study that used medical charts of patients retrospectively. It allowed us to evaluate real clinical data over a longer period. The patients were selected from two private neurology clinics in Amman, the capital of Jordan. Both clinics serve large cities. Most migraine patients are resident in those cities. Clinics were selected based on this criterion. They were chosen because they specialized in treating headache disorders. They also used the same diagnostic criterion for migraine. The data collection took place in March and April 2024. We chose this timeframe to allow for adequate follow-up periods, which are needed to assess treatment outcomes. The study was approved by Yarmouk University IRB (094/2024). The clinicians obtained consent from the patients to use their data for scientific purposes, and all data were kept anonymous.

### 2.2. Inclusion Criteria

Only females, using desvenlafaxine (Davlex^®^, United pharmaceuticals, Amman, Jordan) for at least one month, aged above 18 years, diagnosed with migraine after detailed medical history and neurological examination, and with normal brain MRI were included in the study. The exclusion criteria included: pregnancy, lactation, and other conditions linked to chronic pain. It also included treatment with other antidepressants or migraine medications. The study duration was six months. Participants recorded migraine frequency, intensity, and duration in headache diaries. Desvenlafaxine’s efficacy was measured by MIDAS before starting therapy. It was measured again at the end of the study.

### 2.3. Study Instrument

A well-structured study instrument was created to collect information about the patients. The demographics comprised age, smoking status, and employment status. The clinical information included family history of migraine, the presence or absence of any comorbidities with migraine, the duration of migraine diagnosis, migraine headache type (episodic, or chronic), brain MRI, and the dose and duration of Davlex^®^.

### 2.4. Outcome Measurement

Migraine severity was the primary outcome measure of the study. This was evaluated using five structured questions, as in previous literature [34]: Number of migraine days per month, the average headache duration in minutes, Headache severity visual analog scale (0–10), the use of acute medication, and the frequency of acute medication use per week. These measures were taken at the baseline and at the follow-up visits by the therapist.

### 2.5. Data Analysis

Descriptive data of the demographic and clinical data were depicted for each of the 10 cases. In addition, the paired *t*-test was used to examine the difference in the mean scores of the outcome variable (migraine) between the baseline and the follow-up visit. Confidence intervals were set at 95% and significance at *p* < 0.05. Data were presented as mean ± Standard deviation (SD). Data were analyzed using SPSS software version 21.

## 3. Results

### 3.1. Study Sample Characteristics

Data were analyzed from 10 female patients diagnosed with migraine. The patients’ ages ranged between 22 and 50 years old. Nine out of ten patients were non-smokers, eight were unemployed, six did not have a family history of migraine, and all the patients reported normal brain MRI. The collected data on patient histories included migraine frequency and severity and response to treatment. It also considered potential triggers. These included anxiety and depression. This provided a more comprehensive understanding of the patient’s migraine profile. Please refer to Table 1 for detailed patient information.

### 3.2. Migraine Assessment

Table 2 provides a detailed description of the number of migraine days per month, headache duration in minutes, headache severity visual analog scale (0–10), the required acute medication, the frequency of acute medication use per week, and the side effects of each of the cases. Parameters such as these give the trend of migraine attacks and treatment efficacy. It also shows variations in how individuals respond to the laid-down strategies. These strategies are for managing migraines. This information is vital as it is used to tailor treatment plans for better patient outcomes.

Table 3 demonstrates the paired *t*-test analysis to compare the migraine severity at baseline and the follow-up visit after using desvenlafaxine. For example, the number of migraine days per month at baseline (14.70 ± 3.68) was significantly reduced at the follow-up (2.50 ± 2.50), ((t = 8.54, 9), *p* < 0.001). Also, headache duration in minutes at baseline (131.25 ± 32.81) was significantly reduced at the follow-up (52.50 ± 44.64) ((t = 4.20, 7), *p* = 0.004). Headache severity visual analog scale at baseline (6.80 ± 0.63) was reduced significantly at the follow-up (2.70 ± 2.40) ((t = 5.16, 9), *p* = 0.001)), and the frequency of acute medication use per week at baseline (3.30± 1.49) was significantly reduced at the follow-up (0.80 ± 0.92), ((t = 7.32, 9), *p* < 0.001). This is a significant improvement in the syndrome of migraines. It also cuts the need for acute medication after using desvenlafaxine. The statistical analysis is significant. It shows that desvenlafaxine reduces migraines’ frequency, duration, and severity.

In summary, the results demonstrate that desvenlafaxine was able to significantly reduce all the outcome variables investigated, namely, the number of migraine days per month, the average headache duration in minutes, the headache severity on the visual analog scale, and the frequency of acute medication use per week.

## 4. Discussion

Migraine is a widespread disorder featuring moderate to severe unilateral pain episodes that can last from 4 h to 3 consecutive days. This is often accompanied by other symptoms such as nausea, vomiting, pallor, fatigue, lack of concentration, photophobia, diarrhea, and others [42]. The pathophysiological basis of migraine pain is neurogenic inflammation and vasodilation in the meninges, which leads to the sensitization of the nociceptive afferents [43]. While acute analgesics are recommended for all people with migraine, use of prophylaxis is an essential strategy for several patients. This study tried to explore the potential of using desvenlafaxine in migraine prophylaxis to provide clinicians with another therapeutic option that can extend the list of available prophylactic medications. Our results from this small-scale study of 10 cases demonstrated promising results. Desvenlafaxine was able to diminish the number of migraine days per month, the average headache duration in minutes, the headache severity on the visual analog scale, and the frequency of acute medication use per week.

According to an evidence-based comprehensive review [36], the prophylactic therapies are grouped according to their postulated efficacy and side effects as follows: Group 1: moderate to highly effective with infrequent side effects, including amitriptyline and valproate. Group 2: Lower efficacy than group 1, and mild to moderate side effects, including aspirin, gabapentin, and atenolol. Group 3: effective prophylactic medications that lack substantial evidence, such as bupropion and diltiazem. Group 4: Medium to high efficacy medications, good strength of evidence, but with side effect concerns, such as flunarizine. Group 5: medications with no efficacy in migraine prophylaxis, such as carbamazepine.

The ultimate goal of prophylactic therapy is to both reduce the number and the severity of the episode and therefore improve the quality of life of the patients and enhance their daily functioning. Recently, besides the clinical efficacy, patient satisfaction and willingness to start prophylactic therapy have also been taken into consideration [1,42,44].

Researchers believe that desvenlafaxine provides extensive relief for migraine sufferers. This is because it cuts migraine days. It also reduces the length and severity of headaches and it lowers the use of acute medication. Improvements in all these measures show desvenlafaxine’s potential to improve patients’ lives. It also reduces the burden of migraines.

Our finding is consistent with the only available previous study [40], where desvenlafaxine demonstrated efficacy in migraine prophylaxis. Although the entire mechanism is not fully understood, based on existing evidence, this effect has been attributed to the 5-HT and NE reuptake mechanism of desvenlafaxine, which is the same mechanism found in venlafaxine and amitriptyline, the famous tricyclic antidepressant [45,46,47]. Clinical experience supports this explanation. Amitriptyline has been used for chronic pain for decades, and it has also been employed in migraine prophylaxis [48,49]. Desvenlafaxine acts on serotonin and norepinephrine. This may also help it work to prevent migraines. Serotonin helps to modulate pain and regulate blood vessel tension, while norepinephrine helps with pain perception and autonomic nervous system function. Desvenlafaxine may stabilize migraine-related neural pathways by modulating key neurotransmitter systems.

On the other hand, due to the side effects profile of amitriptyline, which includes cardiovascular side effects, sedation, postural hypotension, dry mouth, and histaminergic effects [50,51], its use has declined and it has been replaced by venlafaxine, a newer SNRI medication that shares the same mechanism but comes with higher tolerability [52].

Desvenlafaxine, the principal metabolite of venlafaxine, is understudied in migraine prophylaxis. Venlafaxine, however, has been extensively studied in migraine prophylaxis. For example, venlafaxine was shown to decrease the mean number of headaches per month starting from the first month of treatment and reaching maximum effects in 6–7 months of use. Venlafaxine also improved the reported global efficacy in about 88% of patients with migraine [53,54]. The efficacy of venlafaxine is dose-related as higher doses ensure higher inhibition of neuronal NE reuptake [54]. However, according to some studies, about 18% of patients discontinued venlafaxine due to side effects that could be dose-related [53]. In our study, desvenlafaxine worked as well as venlafaxine did in past studies. However, its simpler metabolism and once-a-day dosing may make desvenlafaxine more effective. It may lead to better patient compliance and less potential for drug-drug interactions. More studies are needed to compare venlafaxine and desvenlafaxine for preventing migraines. These studies will help us understand their effectiveness and tolerability. Desvenlafaxine’s effects on migraine would have been outside of serotonin and norepinephrine’s influence. For example, it could ease the cortical spreading of depression. This is a key part of migraine with aura. Additionally, it could block neurogenic inflammation and the influence of calcitonin gene-related peptides. These have been linked to migraine pain. Research into these supposed mechanisms may better explain their role.

Migraine prophylaxis research is beset by challenges in different aspects.

The main challenge in the investigation of desvenlafaxine’s role in migraine prophylaxis is the lack of official approval. As such, the derivation of clinically relevant data must be obtained from retrospective studies such as the current investigation, as well as from consensus and the personal experience of neurologists that used desvenlafaxine to control depressive symptoms in people with migraine. In general, the diagnosis of headache is challenging; for example, migraine symptoms could be mediated by the medication overuse headache (MOH) that could interfere with accurate migraine subtype diagnosis. MOH does not exclude a diagnosis of refractory migraine. The present study recruited data from females diagnosed with migraine. According to evidence, the risk of MOH is higher in females, especially those residing in developing countries or with low socioeconomic status [55,56,57]. MOH diagnosis can be easily confirmed by the withdrawal of medications leading to improvement in headache [58].

Another major clinical challenge in migraine assessment is the presence of comorbid pain conditions. According to one study, 51% of the patients with migraine reported having one or more comorbid painful conditions; this percentage rose to 70% in patients diagnosed with chronic migraine [59]. For example, fibromyalgia has been reported in around 30% of patients with migraine [59]. Furthermore, migraine is closely associated with psychological distress; one study underscored that depression, anxiety, and insomnia are among the non-painful conditions associated with migraine [60]. This could be an opportunity to use antidepressants such as desvenlafaxine to manage anxiety and depressive symptoms while keeping an eye on the possible improvement in migraine burden. Another challenge in migraine, besides the cultural and socioeconomic factors, is the educational competencies among healthcare practitioners in developing countries. Although headache symptoms are one of the most commonly reported in clinics, in some countries it is overlooked. This could be linked to the poor educational and professional services offered to these patients [61]. For example, in one study among neurology residents involving >200 participants, about 30% self-reported difficulties in diagnosing migraine, and the most reported barrier was the accurate communication and collaboration between the patient and the healthcare provider [62].

Our findings showed that desvenlafaxine was well-tolerated as only one case reported constipation at 100 mg dose, and this side effect subsided after dose reduction to 50 mg. This result is in line with the existing literature, which underscores the high tolerability profile of desvenlafaxine. However, dry mouth and constipation were reported in previous studies at the 50 mg and the 100 mg doses [63]. In addition, desvenlafaxine could be associated with vasoconstriction syndrome and severe headaches [64]. Besides its tolerability in general, desvenlafaxine’s preferable pharmacokinetics, primarily its lower incidence of inhibiting the hepatic enzyme Cytochrome P450, which minimizes the chance of drug interactions, makes it an attractive choice for clinicians [38].

This work adds to the very little existing literature examining the role of desvenlafaxine in migraine prophylaxis, especially in women. However, the limited study sample prevents the generalization of the results. Therefore, future larger-scale follow-up studies are required to fully elucidate the potential efficacy of desvenlafaxine in migraine attack prevention. This is a small study using retrospective data. As such, the results are very preliminary. The study has a small sample size and is non-randomized. These are significant limitations for drawing general conclusions. Additionally, this was not a blinded and controlled trial. It did not rule out the placebo effect. It also did not rule out changes in migraine frequency. Larger, prospective, randomized controlled trials should address these limitations in the future. They could also investigate whether desvenlafaxine is effective for different types of migraine. They could also assess its long-term effectiveness and safety and could also compare it directly to other established prophylactic treatments. In short, this preliminary study analyzed data retrospectively from ten women with migraine using validated tools in its assessment and included clinical details of the patients’ points facilitating the performance of larger-scale studies of the role of desvenlafaxine in migraine prophylaxis. The reported findings show an improvement in all the assessment parameters under investigation, namely, the number of migraine days per month, the average headache duration in minutes, the headache severity on the visual analog scale, and the frequency of acute medication use per week.

## 5. Conclusions

This study provides early preliminary evidence that supports the use of desvenlafaxine to prevent migraines in female patients. It may improve life quality and reduce the burden of migraines for patients. This fits with desvenlafaxine inhibiting both serotonin and norepinephrine reuptake. Desvenlafaxine was very well tolerated in our group. Only one case of mild side effects was noted. Although not conclusive, the results are promising. However, interpreters must approach the study’s limitations with caution. The first limitation is its small sample size. The second is its retrospective nature. This work makes some discoveries. They add to very poor existing literature about desvenlafaxine in migraine prevention. They suggest it may be a valuable addition to the drugs for managing migraine, and that females with depression and migraine could benefit from desvenlafaxine. More research is required. It should be larger and prospective. The studies should be randomized and controlled. These studies need to fully demonstrate how well desvenlafaxine prevents migraines. They are also needed to show its place in clinical practice.

## Figures and Tables

**Table 1:** The description of the patient’s demographics and clinical information.

	Age (Years)	Smoking Status	Employment Status	Chronic Conditions	Family History of Migraine	Migraine Diagnosis Since	Migraine Type	Normal Brain MRI
Case 1	49	Non-smoker	Unemployed	Anemia	No	5 years	Chronic	Yes
Case 2	35	Non-smoker	Unemployed		Yes	15 years	Episodic	Yes
Case 3	29	Non-smoker	Unemployed	Anxiety	No	3 years	Episodic	Yes
Case 4	46	Non-smoker	Unemployed		No	12 years	Episodic	Yes
Case 5	50	Non-smoker	Unemployed	Type II diabetes, fibromyalgia	No	3 years	Chronic	Yes
Case 6	50	Non-smoker	Unemployed	Hypertension	No	2 years	Chronic	Yes
Case 7	42	Non-smoker	Employed		Yes	10 years	Chronic	Yes
Case 8	49	Non-smoker	Employed	Shoulder Arthritis	Yes	4 years	Chronic	Yes
Case 9	45	Smoker	Unemployed		No	8 years	Chronic	Yes
Case 10	22	Non-smoker	Unemployed		Yes	6 months	Chronic	Yes

**Table 2:** Migraine assessment at baseline and follow-up visit per each case.

Cases on DSV (Desvenlafaxine)	Time Points	Number of Migraine Days per Month	Headache Duration in Minutes	Headache severity Visual Analogue Scale (0–10)	Required Acute Medication	Frequency of Acute Medication Use/Week	Side Effects Reported
Case 1: DSV 50 mg for 2 months	Baseline	16	120	7	Yes	5	
	Follow-up	5	90	4	Yes	2	
Case 2: DSV 100 mg for 1 month	Baseline	12	120	6	Yes	2	
	Follow-up	0	0	0	No	0	
Case 3: DSV: maintenance 100 mg for 5 months	Baseline	12	180	6	Yes	5	
	Follow-up	4	90	6	Yes	1	
Case 4: DSV: maintenance 100 mg for 1 month	Baseline	10	120	7	Yes	4	
	Follow-up	5	90	4	Yes	2	
Case 5: DSV: maintenance 100 mg for 3 months	Baseline	15	120	7	Yes	2	
	Follow-up	6	90	5	Yes	1	
Case 6: DSV: maintenance 100 mg for 10 months	Baseline	20	180	7	Yes	4	
	Follow-up	0	0	0	None	0	
Case 7: DSV maintenance 100 mg for 3 months, returned to 50 mg	Baseline	17	120	6	Yes	2	Constipation on 100 mg
	Follow-up	1	60	4	None	0	
Case 8: DSV maintenance 100 mg for 12 months, returned to 50 mg	Baseline	15	90	7	Yes	1	
	Follow-up	0	0	0	None	0	
Case 9: DSV maintenance 100 mg for 16 months	Baseline	20	Almost all day long	8	Yes	5	
	Follow-up	4	120	4	Yes	2	
Case 10: DSV maintenance 50 mg for 6 months	Baseline	10	Almost all day long	7	Yes	3	
	Follow-up	0	0	0	0	0	

**Table 3:** Paired-*t*-test analysis for the migraine outcome measurements.

	Baseline Mean ± SD	Follow-Up Mean ± SD	t, df	*p*-Value
Number of migraine days per month	14.70 ± 3.68	2.50 ± 2.50	8.54, 9	<0.001 *
Headache duration in minutes	131.25 ± 32.81	52.50 ± 44.64	4.20, 7	0.004 *
Headache severity visual analogue scale (0–10)	6.80 ± 0.63	2.70 ± 2.40	5.16, 9	0.001 *
Frequency of acute medication use/week	3.30 ± 1.49	0.80 ± 0.92	7.32, 9	<0.001 *

SD: standard deviation, t: *t*-test score, df: degrees of freedom, * *p* < 0.05.

## Data Availability

All data related to this manuscript will be available from the corresponding author upon request.
